# Magnetic resonance imaging in the assessment of hip arthroplasty complications

**DOI:** 10.1590/0100-3984.2015.48.5e2

**Published:** 2015

**Authors:** Flavia Martins Costa

**Affiliations:** 1 Titular Member, Colégio Brasileiro de Radiologia e Diagnóstico por Imagem (CBR), Master, Fellow PhD degree in Radiology at Universidade Federal do Rio de Janeiro (UFRJ), MD, Radiologist, Clínica de Diagnóstico por Imagem (CDPI/DASA), Rio de Janeiro, RJ, Brazil. E-mail: flavia26rio@hotmail.com.

Hip arthroplasty represents one of the greatest technological developments of the modern medicine that is beneficial to a wide range of patients. However, like every surgery, it is subjected to a series of complications, some of them easily and other not so easily recognizable, as in the case of inflammatory pseudotumor.

Pseudotumors may be found in cases of well-functioning hip prostheses as well as in painful hips, and for this reason a clinical evaluation associated with a correct diagnosis are essential both for the prognosis and for the definition of the therapeutic approach to the patients, since the presence of a periprosthetic cystic collection is not necessarily connected with a revision of the prosthesis^(1)^.

In such cases, magnetic resonance imaging (MRI) has shown to be effective and accurate principally in the assessment of soft parts. However, after reading the review article published by Vilas Boas et al.^(2)^ in the present issue of **Radiologia Brasileira**, one can notice that this diagnosis practically represents a challenge to this clinical specialty.

Firstly, the presence of magnetic susceptibility artifacts caused by the prostheses generates great image distortion which limits the appropriate visualization and, consequently, makes the diagnosis more difficult. Despite the availability of innumerable techniques and sequences to reduce such artifacts, varying according the equipment, many times they determine loss of images resolution^(3)^.

Once the technical issues are overcome, there are still the main differential diagnoses to be faced, namely, sarcomas (1% of malignant tumors) that hardly can be recognized, truly a wolf in sheep's clothing, and also abscesses^(2)^. In the diagnosis of the latter, besides a detailed clinical history, diffusion-weighted MRI shows to be extremely effective, but due to prostheses-related technical limitations, it cannot be performed.

Finally, the lack of personal contact between physicians and patient in addition to an incomplete or inappropriately collected clinical history result in failure to detect such a surgical complication.

Thus, radiologists should be quite attentive and recognize that innumerable variables contribute to failure in the early diagnosis of inflammatory pseudotumors. Medical wisdom, besides clinical attention towards the patient and, obviously, technical skills are fundamental in such cases. Reaching this goal will be possible with a technical team constituted of skilled MRI technologists and nursing technicians, besides experienced radiologists with appropriate knowledge.

## References

[1] Chang EY, McAnally JL, Van Horne JR (2012). Metal-on-metal total hip arthroplasty: do symptoms correlate with MR
imaging findings?. Radiology.

[2] Vilas Boas RMS, Madeira IA, Lopes AA (2015). Pseudotumor inflamatório do quadril: uma
complicação da artroplastia a ser reconhecida pelo
radiologista. Radiol Bras.

[3] Yanny S, Cahir JG, Barker T (2012). MRI of aseptic lymphocytic vasculitis-associated lesions in
metal-on-metal hip replacements. AJR Am J Roentgenol.

